# Anti-drug resistance, anti-inflammation, and anti-proliferation activities mediated by melatonin in doxorubicin-resistant hepatocellular carcinoma: in vitro investigations

**DOI:** 10.1007/s00210-023-02385-w

**Published:** 2023-01-18

**Authors:** Ahmed R. Hamed, Shaymaa M. M. Yahya, Heba K. Nabih

**Affiliations:** 1 Chemistry of Medicinal Plants Department, and Biology Unit, Central Laboratory for Pharmaceutical and Drug Industries Research Institute, National Research Centre, 33 El-Bohouth St, Dokki, Giza, 12622 Egypt; 2 Hormones Department, Medicine and Clinical Studies Research Institute, and Stem Cell Lab, Centre of Excellence for Advanced Sciences, National Research Centre, 33 El-Bohouth St, Dokki, Giza, 12622 Egypt; 3 Medical Biochemistry Department, Medicine and Clinical Studies Research Institute, National Research Centre, 33 El-Bohouth St, Dokki, Giza, 12622 Egypt

**Keywords:** Multidrug resistance, Melatonin, Doxorubicin, p53, Anti-inflammation, qRT-PCR, Antibody array

## Abstract

Hepatocellular carcinoma (HCC) is the major life-threatening primary liver malignancy in both sexes all over the world. Unfortunately, the majority of patients are diagnosed at later stages because HCC does not elicit obvious symptoms during its early incidence. Consequently, most individuals escape the first-line HCC treatments and are treated with chemotherapy. Regrettably, the therapeutic outcomes for those patients are usually poor because of the development of multidrug resistance phenomena. Furthermore, most anti-HCC therapies cause severe undesired side effects that notably interfere with the life quality of such patients. Accordingly, there is an important need to search for an alternative therapeutic drug or adjuvant which is more efficient with safe or even minimal side effects for HCC treatment. Melatonin was recently reported to exert intrinsic antitumor activity in different cancers. However, the regulatory pathways underlying the antitumor activity of melatonin are poorly understood in resistant liver cells. Furthermore, a limited number of studies have addressed the therapeutic role of melatonin in HCC cells resistant to doxorubicin chemotherapy. In this study, we investigated the antitumor effects of melatonin in doxorubicin-resistant HepG2 cells and explored the regulatory pivotal targets underlying these effects. To achieve our aim, an MTT assay was used to calculate the 50% inhibitory concentration of melatonin and evaluate its antiproliferative effect on resistant cells. Additionally, qRT-PCR was used to quantify genes having a role in drug resistance phenotype (ABCB1, ABCC1, ABCC2, ABCC3, ABCC4, ABCC5, and ABCG2); apoptosis (caspases-3, and -7, Bcl2, Bax, and p53); anti-oxidation (NRF2); expression of melatonin receptors (MT1, MT2, and MT3); besides, programmed death receptor PD-1 gene. The active form of the caspase-3 enzyme was estimated by ELISA. A human inflammatory antibody membrane array was employed to quantify forty inflammatory factors expressed in treated cells. We observed that melatonin inhibited the proliferation of doxorubicin-resistant HepG2 cells in a dose-dependent manner after 24-h incubation time with a calculated IC_50_ greater than 10 mM (13.4 mM), the expression levels of genes involved in drug resistance response (ABCB1, ABCC1, ABCC5, and ABCG2) were downregulated. Also, the expression of caspase-3, Caspase-7, NRF2, and p53 genes were expressed at higher levels as compared to control (DMSO-treated cells). An active form of caspase-3 was confirmed by ELISA. Moreover, the anti-inflammatory effect of melatonin was detected through the calculated fold change to control which was reduced for various mediators that have a role in the inflammation pathway. The current findings introduce melatonin as a promising anti-cancer treatment for human-resistant HCC which could be used in combination with current chemotherapeutic regimens to improve the outcome and reduce the developed multidrug resistance.

**Supplementary Information:**

The online version contains supplementary material available at 10.1007/s00210-023-02385-w.

## Introduction

Hepatocellular carcinoma (HCC) remains one of the most common causes of death worldwide despite the significant options of therapeutic advancements and improved detection methods (Giraud et al. 2021). Unfortunately, 80% of patients with HCC are currently diagnosed at advanced stages of the disease and are not suitable for the optimal treatment modalities of the tumor. Although chemotherapy treatment with traditional cytotoxic agents (5-Fluoracil, doxorubicin, cisplatin, and oxaliplatin), radiation, and targeted therapies are the main approaches for those patients, drug resistance remains a major clinical obstacle to treatment success (Duan et al. 2019).

Drug resistance is a well-commonly known developed phenomenon in which pharmaceutical therapies are tolerated by tumor cells; without any medical response leading to failure of treatment. It can be either intrinsic or acquired through multifactorial, and pleiotropic cellular signals that are simultaneously contributed to this complication (Housman et al. 2014). Drug resistance phenomena could be mediated by mechanisms that include upregulation of drug efflux pumps [Members of the ATP-binding cassette (ABC) transporter family proteins], downregulation of drug uptake, drug inactivation, alteration in the drug target, DNA damage repair, inhibition of apoptotic signals, tumor environment change, acquiring stem-cell like characteristics, autophagy, and development of epithelial-mesenchymal transition (EMT). More than one drug resistance mechanism can be detected in a single cancer type (Haider et al. 2020). These cross-talks of vital links pose significant challenges to a thorough understanding of the signaling networks through investigative research.

**Fig. 1:**
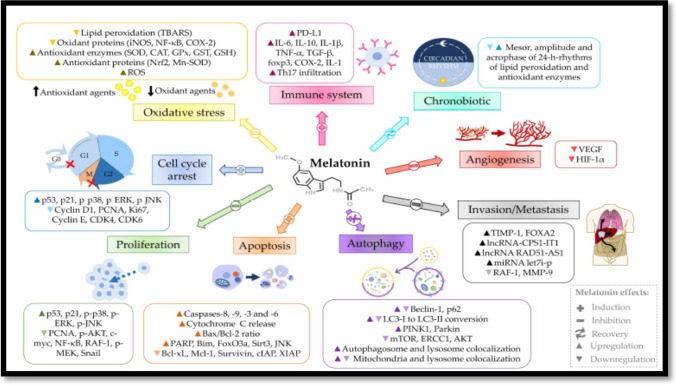
Pathways regulated by melatonin

Treatment with a natural indole amine hormone, melatonin (N-acetyl-5-methoxytryptamine), which is mainly produced by the pineal gland in response to darkness, is receiving increasing attention for cancer management and cure nowadays. This returns to the confirmed properties of melatonin such as antioxidant enzymes regulator, free radical scavenger/production inducer, anti-inflammatory, immunomodulatory agent, angiogenesis suppressor, proliferation, and metastasis inhibitor, apoptosis activator, cell cycle arrest (cell growth reducer), autophagy modulator (both induction, and inhibition; according to the environment of cellular context) (Fig. 1) (Mehrzadi et al. 2021; Fernández-Palanca et al. 2021). Different effects of melatonin may be related to the type of cancer cells, the applied dose of melatonin, and if melatonin entrance is receptor-dependent (MT1 and MT2) or independent (Gurunathan et al. 2021). These all effects recommend melatonin to be a good candidate that uses alone or in combination with anticancer therapies to improve conventional therapies and reduce their undesired side effects (Talib et al. 2021).

Although several oncological studies have been conducted to indicate the anticancer effects of melatonin alone or in combination with chemo-, radiotherapies, this is the first study that assesses the anti-drug resistance activities of melatonin in HCC cell line acquiring doxorubicin resistance. The current research study can help make decisions for the clinical application of melatonin as a combination therapy with chemotherapeutic treatments in the future. This step will aid in giving priority for melatonin to be used as a co-adjuvant agent to chemotherapies for improving their therapeutic effects and ameliorating their toxicity.

CAT = catalase, CDK = cyclin-dependent kinase, cIAP = cellular inhibitor apoptotic proteins, COX-2 = cyclooxygenase-2, ERCC1 = DNA excision repair cross complementary 1 protein, ERK = extracellular signal-regulated kinase, FOXA2 = forkhead box A2, FoxO3a = forkhead box protein O3, foxp3 = forkhead box P3, GPx = glutathione peroxidase, GSH = reduced glutathione, GST = glutathione S-transferase, HIF-1α = hypoxia-inducible factor 1α, IL-1 = interleukin-1, IL-1β = interleukin 1 beta, IL-6 = interleukin-6, IL-10 = interleukin-10, iNOS = inducible nitric oxide synthase, JNK = c-Jun N-terminal kinase 1, LC3 = microtubule-associated protein 1 light chain 3, lncRNA = long non-coding RNA, MEK = MAPK/ERK kinase 1, MMP-9 = matrix metalloproteinase 9, Mn-SOD = manganese superoxide dismutase, mTOR = mammalian target of rapamycin, NF- κB = nuclear factor-kappa B, Nrf2 = nuclear erythroid 2-related factor 2, PARP = poly(ADP-ribose) polymerase, PCNA = proliferating cell nuclear antigen, PD-L1 = programmed death ligand 1, PINK1 = PTEN-induced putative kinase 1, RAF-1 = ras-activated factor 1, ROS = reactive oxygen species, Sirt3 = sirtuin 3, Snail = zinc finger protein SNAI1, SOD = superoxide dismutase, TBARS = thiobarbituric acid reactive substances, TGF-β = transforming growth factor β, Th17 = IL-17-producing T helper, TIMP-1 = tissue inhibitor of metalloproteinases 1, TNF-α = tumor necrosis factor alpha, VEGF = vascular endothelial growth factor, XIAP = cellular and X-linked inhibitor apoptotic proteins (Fernández-Palanca et al. 2021).

## Materials and methods

### Cell line and cell culture

The wild HepG2 cell line was purchased from ATCC (American Type Culture Collection). These cells were cultured and propagated in 75 cm^2^ flasks in DMEM (Dulbecco’s Modified Eagle Medium; Corning, USA); supplemented with 10% Fetal Bovine Serum (Invitrogen, USA), 1% Penicillin–streptomycin (Invitrogen, USA) and 4 mM L-glutamine (Invitrogen, USA) at 37 °C in a humidified 5% CO2 incubator. Doxorubicin-resistant HepG2 cells (HepG2/dox) were established as previously described by drug selection through a gradual increase of the Doxorubicin dose (Adricin, Hikma Pharmaceuticals, Egypt) in culture until a resistant cell phenotype that over-expresses P-glycoprotein (ABCB1) is obtained (Yahya et al. 2016; Hamed et al. 2018).

### MTT viability assay

HepG2/dox-resistant cells were seeded onto 96-well plates (1 × 10^4^ cells/well) for 24 h and then treated with 0–10 (0.00, 0.02, 0.004, 0.08, 0.16, 0.31, 0.63, 1.25, 2.50, 5.00, and 10.00) mM melatonin (Sigma Aldrich, St. Louis, MO, USA) dissolved in 0.5% Dimethyl sulfoxide (DMSO) (Molecular Biology Grade; Serva, Germany) with an incubation time of 24 h. Cell viability was measured using MTT [3-(4,5-dimethylthiazol-2-yl)-2,5-diphenyltetrazolium bromide] colorimetric assay. MTT (Serva, Germany) working solution (5 mg/ml) was added to each well and incubated at 37 °C for 90 min. After that, DMSO (100 µl/well) was added to dissolve the formazan crystals with shaking for 10 min. The optical density of each well was measured using a microplate reader (Tristart lb 942 microplate reader, Berthold, Germany) at 492 nm against a blank (no cells). Cell viability was calculated as the percentage of viable cells in the melatonin-treated cells versus the untreated control cells. The concentration of the compound that decreased cell viability by 50% cytotoxic concentrations (IC_50_) was calculated from the cell viability curve using fitting into a non-linear regression equation on Prism 8 (GraphPad Software Inc; San Diego, CA, USA) (Bennukul et al. 2014).

### QRT-PCR for genes expression quantification

HepG2/dox-resistant cells were seeded onto 6-well plates (3 × 10^5^ cells/well). After treatment with 13.4 mM melatonin (the calculated IC_50_) for 24 h, total RNA was extracted with Qiazol Reagent (Qiagen, Germany). Genes expression levels were amplified and quantified by qRT-PCR (MiniOpticon Real-Time PCR System, Bio-Rad, France) using SensiFAST SYBR No-ROX one-step kit (Bioline, USA) (Hermyt et al. 2019) in a final volume of 15 µl containing 7.5 µl 2 × sensiFAST SYBR No-ROX mix, 0.3 µl Ribosafe RNase Inhibitor, 0.15 µl Reverse transcriptase, 3.85 µl H_2_O, 2 µl RNA sample with specific primer pairs (0.6 µl each), as indicated in Table 1. The PCR cycle steps were the following: one cycle for reverse transcription at 45 °C for 10 min, then one cycle for polymerase activation at 95 °C for 2 min, then 40 cycles for both denaturation (at 95 °C for 5 s), and annealing/extension (at 60 °C for 20 s). All RT-PCRs were performed in duplicate, and the gene copy numbers were normalized to 100,000 copies of the housekeeping beta-actin gene.

**Table 1:** RT-PCR primer sequences

Gene	Function	Primer sequence
β-actin (37 Kb)	Housekeeping	**(F)**5′-CCTTCCTGGGCATGGAGTCCT-3′ **(R)**5′-GGAGCAATGATCTTGATCTTC-3′
ABCB1 (210 Kb)	Drug efflux pump	**(F)**5′-AGACATGACCAGGTA-TGCCTAT-3′ **(R)**5′-AGCCTATCTCCTGTCGCATTA-3′
ABCC1 (194 Kb)	Drug efflux pump	**(F)**5′-CATTCAGCTCGTCTTGTCCTG-3′ **(R)**5′-GGATTAGGGTCGTGGATGGTT-3′
ABCC2 (69 Kb)	Drug efflux pump	**(F)** 5′-CCTGGAAGATGTTGAAAAGAAAA-3′ **(R)** 5′-CTGAAGGCAGAAAGACTGAATGA-3′
ABCC3 (57 Kb)	Drug efflux pump	**(F)** 5′-TTTTCTGGTGGTTCACAAAG-3′ **(R)** 5′-GATCTGTCCTCTTCCTTTAG -3′
ABCC4 (281 Kb)	Drug efflux pump	**(F)** 5′-GGCAGTGACGCTGTATGG-3′ **(R)** 5′-CGCCAGGTCTGACAGTAAAG-3′
ABCC5 (98 Kb)	Drug efflux pump	**(F)** 5′-AGAGGTGACCTTTGAGAACGCA-3′ **(R)** 5′-CTCCAGATAACTCCACCAGACGG-3′
ABCG2 (66 Kb)	Drug efflux pump	**(F)**5′-GCGACCTGCCAATTTCAAATG-3′ **(R)**5′-GACCCTGTTAATCCGTTCGTTT-3′
Caspase-3 (22 Kb)	Apoptosis	**(F)**5′-TGGTTCATCCAGTCGCTTTG-3′ **(R)**5′-ATTCTGTTGCCACCTTTCGG-3′
Caspase-7 (51 Kb)	Apoptosis	**(F)**5′-GGAGAAAGCTCATGGCTGTGT-3′ **(R)**5′-TCCCCTTGGCTGTGTTTTG-3′
Bcl-2 (196 Kb)	Anti-apoptosis	**(F)** 5′- GATTGTGGCCTTCTTTGAG-3′ **(R)** 5′-CAAACTGAGCAGAGTCTTC-3′
Bax (6.9 Kb)	Apoptosis	**(F)** 5′- GTTTCATCCAGGATCGAGC-3′ **(R)** 5′-GCCGTCAGAAAACATGTCAG **-**3′
P53 (20 Kb)	Apoptosis, Anti-proliferation, and cell cycle arrest	**(F)** 5′-TGCGTGTGGAGTATTTGGATG-3′ **(R)** 5′-TGGTACAGTCAGAGCCAACCTC-3′
NRF2 (174 Kb)	Antioxidant	**(F)** 5′-CAGCGACGGAAAGAGTATGA-3′ **(R)** 5′-TGGGCAACCTGGGAGTAG-3′
MT1 (22 Kb)	Melatonin Receptor 1A	**(F)** 5′-CGTTGGTGCTGATGTCG-3′ **(R)** 5′-AGTTTGGGTTTGC GGTC-3′
MT2 (15 Kb)	Melatonin Receptor 2	**(F)** 5′-CAACTGCTGCGAGGCG-3′ **(R)** 5′-GGCGGTGGTGA CGATG-3′
MT3(NQO2) (31 Kb)	Melatonin Receptor 3	**(F)** 5′-GGAACCCAAGTCTTTCAACGG-3′ **(R)** 5′- TGGGCTCTTCCTTCCAGATGG -3′
PD-1 (9 Kb)	Programmed death receptor	**(F)** 5′**-**CAGGGTGACAGAGAGAAGGG-3′ **(R)** 5′**-**CCTGGCTCCTATTGTCCCTC-3′

### Human inflammation antibody array for quantitative detection of melatonin effect in an inflammatory pathway

For the simultaneous detection of 40 Human inflammatory factor concentrations in melatonin-treated HepG2/dox-resistant cell lysates, human inflammation antibody array- membrane (cat# ab 134,003, Abcam, USA) was used (Lu et al. 2020). The manufacturer protocol was followed. Briefly, after blocking the membrane array with 1X blocking buffer, the membrane was incubated with the sample lysate (140 µg) overnight at 4 °C. The membrane array was washed thoroughly with wash buffers I, and II that were supplied in the kit. The membrane was then incubated with 1 × Biotin-conjugated anti-cytokines overnight at 4 °C. After washing steps, the membrane was incubated with 1 × HRP (horseradish peroxidase)-conjugated streptavidin for 2 h at RT. The Chemiluminescence signals were detected by a CCD (charged-coupled device) camera of a chemiluminescence imager (UVP, UK) to digitally visualize protein spots on the developed membranes. Spot densitometric analysis of the arrays was performed using Visionworks ls (Analytik Jena, Germany). Then, the background signals were subtracted, and normalization to the positive control was calculated before comparing analyte-by-analyte to determine relative differences in cytokine expression in each sample.

### Detection of active (cleaved) Caspase-3 by ELISA

HepG2/dox-resistant cells were seeded onto 6-well plates (3 × 10^5^ cells/well). After treatment with 13.4 mM melatonin for 24 h, total protein was extracted with RIPA lysis buffer supplemented with a protease inhibitor cocktail (Invitrogen, USA). Total protein concentration was estimated using the BCA protein assay kit (Cat# 23,225; Thermo scientific, USA) by following the manufacturer’s instructions. The protein concentration of each sample was calculated from the Bovine Serum Albumin (BSA) standard curve using Prism 8 (GraphPad Software Inc; San Diego, CA, USA). The diluted protein samples (1:10), and standards were then applied as duplicates to each well of the Human Caspase-3 (active) ELISA Kit (Cat# KHO1091; Invitrogen, USA). By following the methodology steps of the kit, active caspase-3 (ng/mg total protein of lysate) was calculated for each sample using the caspase-3 standard curve (Núñez-Iglesias et al. 2018).

## Statistical analysis

All the mean values and standard error of means were calculated using Microsoft Excel (version of 2007, Microsoft Corporation, USA). All presented data are representative of three independent experiments. Differences between treated cells were analyzed using a student’s *t*-test. *P-*values of < 0.05 were considered significant, and *P-*values of < 0.01 were considered highly significant (indicated by asterisks *, and **, respectively). All figure charts were established with GraphPad Prism version 5.0 (GraphPad Software Inc., San Diego, CA, USA).

## Results

**Fig. 2:**
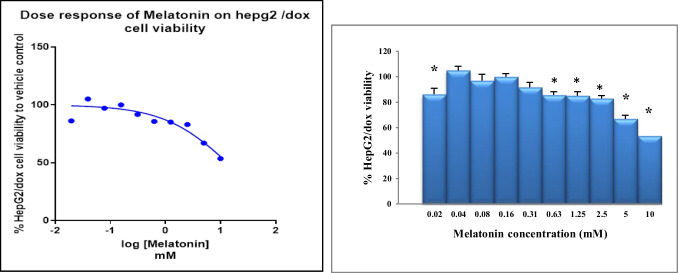
The cytotoxicity curve (left) and histogram (right) show the effect of melatonin on HepG2/dox cell viability after 24 h using MTT assay. HepG2/dox cells were treated with various concentrations of melatonin (0–10 mM). Melatonin was found to reduce 50% viability of resistant cells at a melatonin concentration greater than 10 mM (13.4 mM). The experiment was performed in octuplets

Through MTT assay, we recorded that the cytotoxic effect of melatonin on the viability of HepG2/doxorubicin-resistant cells is time, and dose-course dependent. Melatonin was found to significantly decrease the cell viability of HepG2/dox cells with a calculated IC_50_ of 13.4 mM (> 10 mM). Our current findings suggested that melatonin might be useful as a safe adjuvant in HCC therapy because of its antiproliferative properties (Fig. 2).

**Fig. 3:**
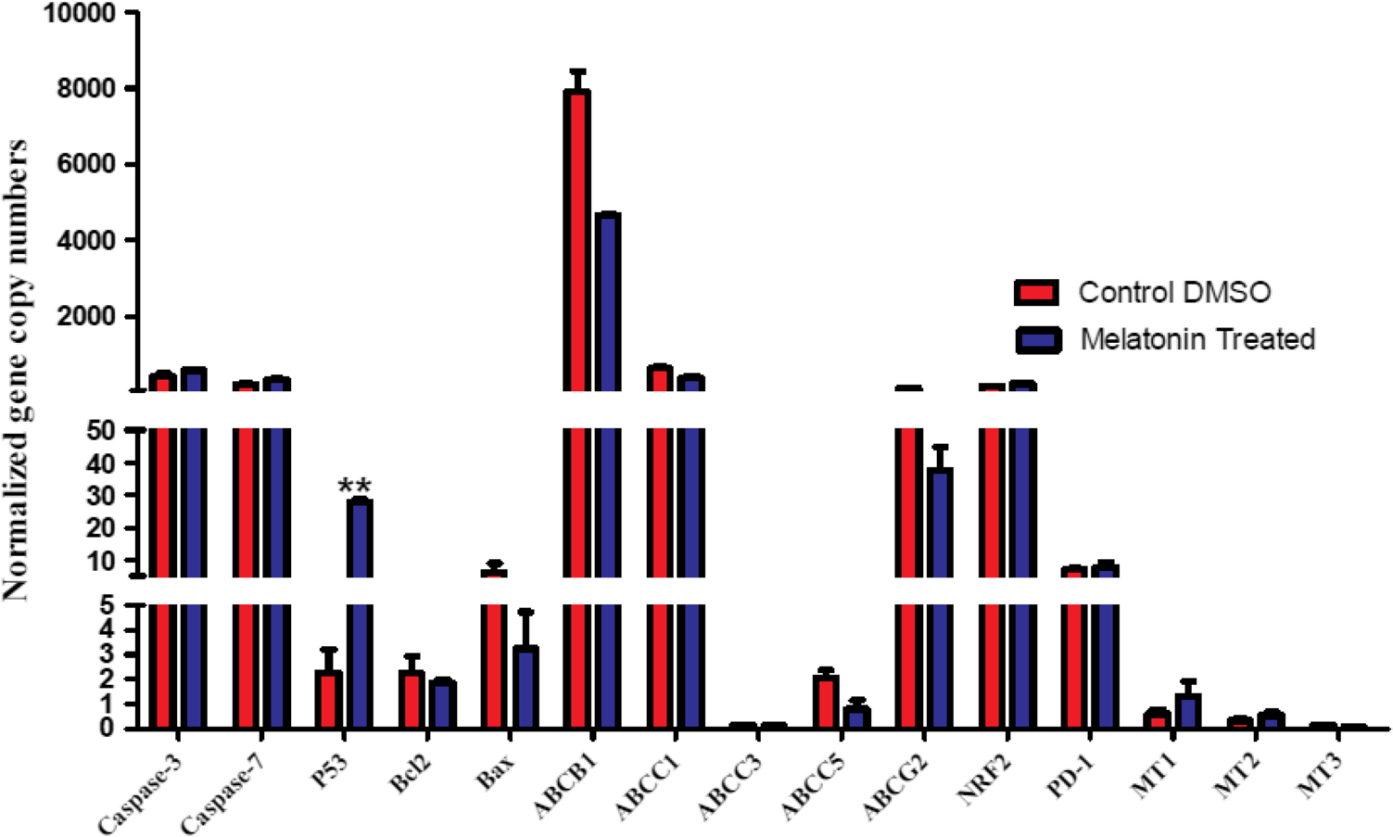
The effect of 13.4 mM melatonin on genes expressions of HepG2/dox cells, showing a highly significant increase in p53 gene expression level (*P* < 0.01)

Through the results of the quantified genes by qRT-PCR, we noticed results indicated that melatonin at IC_50_ dose (13.4 mM) could decrease the expressions of ABCB1, ABCC1, ABCC5, and ABCG2 genes that mediated drug resistance in HepG2/dox cells, as compared to control. Both ABCC2 and ABCC4 did not found to be expressed by these cells. Moreover, the levels of caspase-3, Caspase-7, and NRF2 (nuclear factor erythroid 2–related factor 2) genes were detected to be expressed in slightly but not significantly higher levels as compared to the control. Interestingly, our results recorded a highly significant increase in the genetic expression level of p53 in cells treated with melatonin, in a comparison with control cells. Such increases could force cells to apoptosis, and cell cycle arrest. At the same time, the expression of the anti-apoptotic, Bcl-2 (B-cell lymphoma 2), gene appeared to be quantified at a somewhat lower level (Fig. 3). These data show the impact of melatonin in reducing the undesired side effects of chemotherapy, by relief of drug resistance mechanisms. So, melatonin could be applied as a post/combination therapy for the treatment of human HCC.

**Fig. 4:**
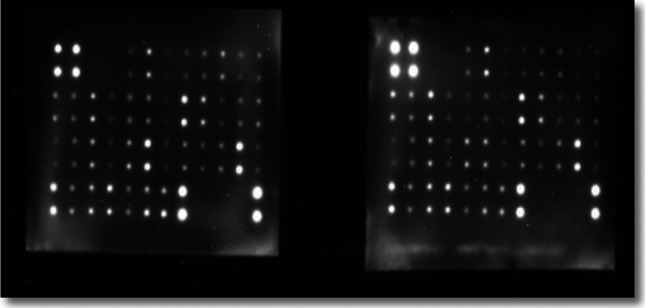
Detection of human 40 inflammatory mediators in doxorubicin-resistant cells using Human inflammation antibody array membrane. Vehicle–treated control HepG2/dox cells (left) versus HepG2 /dox cells treated with 13.4 mM of melatonin (IC_50_ dose) (right)

**Fig. 5:**
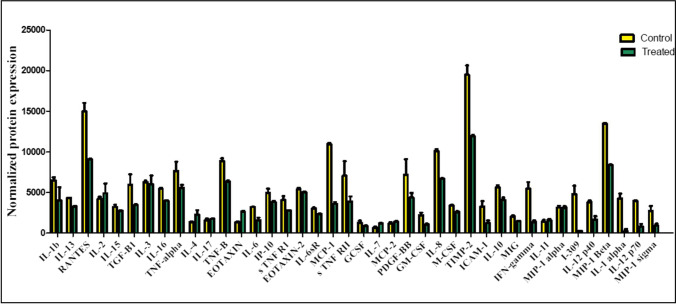
The significant effect of melatonin as an anti-inflammatory mediator in doxorubicin-resistant HCC cells. The data showing the normalized inflammatory proteins expression level means in 13.4 mM melatonin-treated HepG2/dox-resistant cells as a comparison to control (DMSO) HepG2 cells

**Fig. 6:**
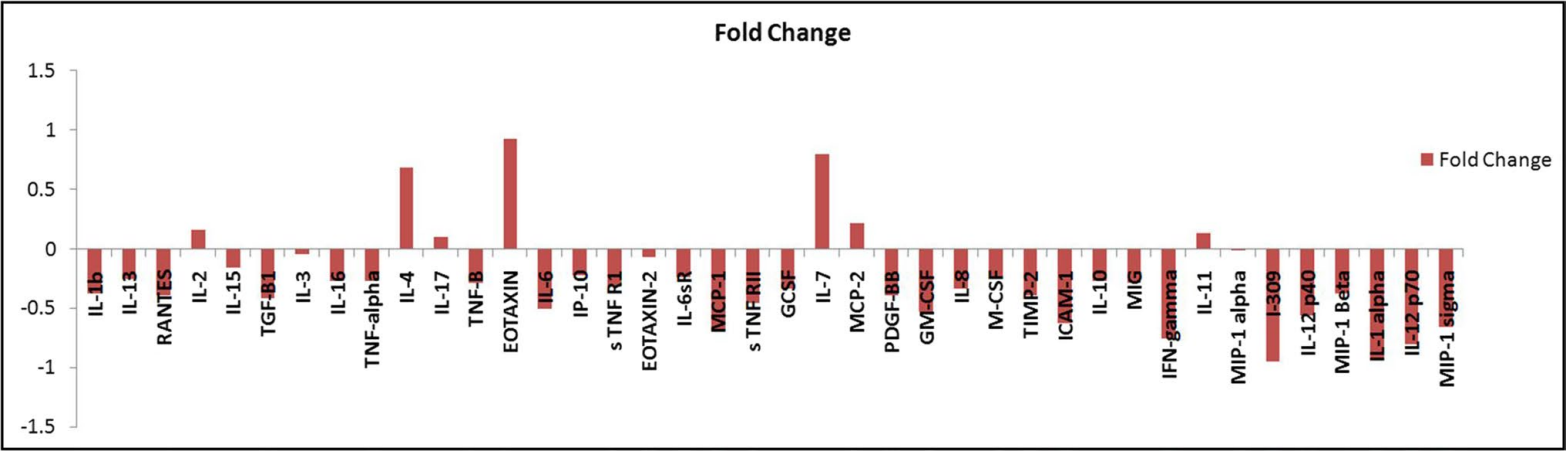
The calculated fold change (of 40 inflammatory factors expressed in 13.4 mM melatonin-treated HepG2/dox-resistant cells) to control

Through the results of the quantitative detection of the melatonin effect in an inflammatory pathway by the Human inflammation antibody array, we confirmed the activity of melatonin as an anti-inflammatory agent that can effectively decrease (with a noticed fold change to control) the expression of 33 significant inflammatory proteins translated in HepG2/dox cells (Figs. 4, 5, 6, and Table 2). At the same time, 13.4 mM of melatonin elevated the expression of the other remnant 7 inflammatory factors including IL-2 (interleukin -2), IL-4, IL-7, IL-11, IL-17, MCP-2 (monocyte chemotactic protein-2), and EOTAXIN (eosinophil chemotactic protein). Interestingly, the increased levels of an anti-cancer cytokine, IL-2, are significant for treating metastatic cancers by inhibiting tumor growth via the activation of lymphocytes (Sun et al. 2018). Additionally, the induced level of IL-7 by melatonin enhances the anti-tumor activity of CD8^+^ T-cells in HCC patients and has been suggested to be a therapeutic candidate for HCC treatment **(**Teng et al. 2018). It has proved that MCP-2 has a role as an inducer for apoptosis in many cell types, such as human aortic smooth muscle cells, and multiple myeloma (Wang et al. 2015); moreover, investigators have proposed that EOTAXIN could suppress tumor growth by mediating a cytotoxic response to cancerous cells (Lotfi et al. 2007). Additionally, Wang et al. 2016a, b suggested that the downregulation of MCP-2 and EXOTAXIN-1 in hepatoma cell lines is associated with the carcinogenesis of liver cells. Meanwhile, IL-17 mediates cancer promotion through induction of IL-6, and accordingly, an activation of IL-8 and both IL-6 and IL-8 were found to be decreased in their expression as a response to 13.4 mM of melatonin treatment. Therefore, IL-17 appears to be a pleiotropic cytokine that acts as a protumor or antitumor activator in cancer development and this action depends on the immunogenicity of tumor models **(**Benchetrit et al. 2002). On the other hand, elevated expression levels of IL-4, and IL-11 in our results were reported to increase the malignancy of liver carcinoma, and drive HCC recurrence, and progression (Zheng et al. 2016; Lauko et al. 2019; Kim et al. 2020).

**Table 2:** Array map showing 40 inflammatory factors in one experiment

	A	B	C	D	E	F	G	H	I	J	K	L
1	Pos	Pos	Neg	Neg	EOTXIN	EOTAXIN-2	GCSF	GM-CSF	ICAM-1	IFN-γ	I-309	IL-1α
2	Pos	Pos	Neg	Neg	EOTAXIN	EOTAXIN-2	GCSF	GM-CSF	ICAM-1	IFN-γ	I-309	IL-1α
3	IL-1b	IL-2	IL-3	IL-4	IL-6	IL-6sR	IL-7	IL-8	IL-10	IL-11	IL-12 p40	IL-12 P70
4	IL-1b	IL-2	IL-3	IL-4	IL-6	IL-sR	IL-7	IL-8	IL-10	IL-11	IL-12 p40	IL-12 p70
5	IL-13	IL-15	IL-16	IL-17	IP-10	MCP-1	MCP-2	M-CSF	MIG	MIP-1α	MIP-1β	MIP-1δ
6	IL-13	IL-15	IL-16	IL-17	IP-10	MCP-1	MCP-2	M-CSF	MIG	MIP-1α	MIP-1β	MIP-1δ
7	RANTES	TGF-β1	TNF-α	TNF-β	S TNF RI	S TNF RII	PDGF-BB	TIMP-2	BLANK	BLANK	Neg	Pos
8	RANTES	TGF-β1	TNF-α	TNF-β	S TNF RI	S TNF RII	PDGF-BB	TIMP-2	BLANK	BLANK	Neg	Pos

Through the detection of active caspase-3 by ELISA, it was confirmed that the HepG2/dox cells treated with 13.4 mM melatonin have an active form of caspase-3 (apoptotic protein) **(**Table 3). This result agrees with the result of gene expression.

**Table 3:** Expression pattern of active caspase-3 in resistant HepG2 cells

	O.D	Total protein concentration (µg/ml)	Concentration of active (cleaved) caspase-3 (ng/ml)	Active caspase 3 (ng/mg total protein of lysate)
(Dox.-resistant cells + 13.4 mM Melatonin)	**0.109**	573.09	0.28	0.495 ± 0.006* (*p* < 0.05)
**(**Dox.-resistant cells + DMSO)	**0.119**	1118.5	0.40	0.358 ± 0.001

* A significance increase as compared to control

## Discussion

Hepatocellular carcinoma (HCC) is the fifth most common cancer that affects liver cells and is considered to be the third among cancer-related deaths worldwide **(**Wang et al. 2017**)**. There are different first-line strategies for HCC treatment, but because of the late diagnosis of the disease which mainly would be associated with additional complications; the therapeutic options are limited to chemotherapy. However, the patient’s quality of life is adversely affected by the severe side effects of most chemotherapeutic agents (Wang et al. 2018). One of the key chemotherapies that were used for HCC treatment is the anthracycline antibiotic, doxorubicin. Doxorubicin can initiate its cytotoxicity through the elevation of reactive oxygen species (ROS) generation, increasing double-strand breaks, and DNA damage, and this will force the cells to be arrested for repair or apoptosis. Unfortunately, the utilization of doxorubicin for HCC treatment is limited with time because of the induced expression of ATP-binding cassette (ABC) transporter protein members which facilitate the efflux transporting of doxorubicin outside the cells (Cox and Weinman 2016).

Melatonin (N-acetyl-5-methoxytryptamine) was recently reported to be a non-toxic indole hormone that exerts oncostatic actions in various cancers. The anti-tumor role of melatonin in hepatic tumors involves a number of different molecular and cellular processes including reduction of cellular proliferation, cell cycle arrest, limiting angiogenesis and metastasis, free radical-scavenging, anti-inflammation, and promoting apoptosis (Su et al. 2017). However, the regulatory pathways underlying the antitumor activity of melatonin are poorly understood. Furthermore, a limited number of studies have addressed the therapeutic role of melatonin in HCC. To our knowledge, this is the first research that discusses and explores the mode of action of melatonin in doxorubicin-resistant HCC cells.

Through our research results, we could expect and confirm that treatment of wild HepG2 cells with increasing doses of doxorubicin aids in increasing the levels of ROS (reactive oxygen species) accumulations, inducing the inflammatory pathway by increasing the levels of proinflammatory cytokines, such as IL-1β (Interleukin-1 beta), IL-6, IL-8, MCP-1 (monocyte chemoattractant protein 1), GM-CSF (Granulocyte–macrophage colony-stimulating factor), IFN-γ (Interferon-gamma), TNF-α (tumor necrosis factor-alpha) (Ho and Piquette-Miller 2006; Kang et al. 2013; Vyas et al. 2014; St. John 2015; Wang et al. 2016a, b) and IL-23 (Hou et al. 2021), and elevating ABCs family members expression (as detected in control vehicle HepG2/dox cells). Upon treating resistant cells with the calculated IC_50_ dose of melatonin (13.4 mM), melatonin will directly pass the membrane in a way that is independent of melatonin receptors (our data detected insignificant expression of melatonin receptor genes with melatonin administration for 24 h) to drive cell differentiation events, reduce cancer cell proliferation, and eliminate invasive properties (Grant et al. 2009), then melatonin will directly/indirectly targeting ABC-transporters; ABCB1, ABCC1, ABCC5, and ABCG2 genes. This finding was supported by one study that illustrated that the reduction of ABCG2 expression was due to the methylation of its promoter by the action of melatonin combination with temozolomide in BTSCs and A172 malignant glioma cells **(**Martín et al. 2013). Additionally, one recent research confirmed the inhibitory effect of melatonin on ABCB1 expression in epirubicin-treated diffuse large B cell lymphoma cells (Liu et al. 2021). Besides, melatonin was discovered to decrease the expression of ABCB1 in melatonin-treated vincristine-resistant oral cancer cell lines via up-regulation of microRNA-34b which targets the ABCB1 gene (Hsieh et al. 2020). Also, Tanoğlu et al. (2021) recorded significantly lower expression of both ABCC5 and ABCG2 genes in melatonin-treated chronic pancreatitis.

Moreover, melatonin will activate the antioxidant transcription factor (Fernández-Palanca et al. 2021; Colares et al. 2022), NRF2, which itself will start the ROS scavenging cascade. So according to our first findings, melatonin appears to act as an anti-drug resistance agent, recover the bad side effects of chemotherapy, could sensitize the cells for anti-tumor drugs, and could be administrated individually, or in combination, or post-chemotherapies as a promising supplementary component that will reduce, and minimize the side-effects of anticancer drugs. This explanation is similar to one study conducted on lung cancer cells A549 and IMR90. They concluded that melatonin does not work only as an anticancer agent, but also protects cells from the adverse conditions caused by doxorubicin. This protective action was initiated by decreasing the doxorubicin-induced ROS levels in a melatonin receptor-independent manner (Song et al. 2012; Zhelev et al. 2017). As melatonin was previously reported in many studies to induce the apoptotic pathway (Javier et al. 2008; Moreira et al. 2015; Ordoñez et al. 2015; Chuffa et al. 2016; Martínez-Campa et al. 2017; Ao et al. 2020; Fernández-Palanca et al. 2021; Das and Samanta 2022; Ammar et al. 2022), it is interestingly to agree with all of these publications by recording in our results the preparatory increasing levels of caspase-3 (both gene, and protein levels), caspase-7, and a highly significant increase in the expression level of p53 gene (*P* < 0.01).

Although melatonin was defined to has anti-inflammatory activities via blocking of pro-inflammatory cytokines, and activating anti-inflammatory interleukins (Chen et al. 2016; Zare et al. 2020), this is the first study that quantifies forty inflammatory factors mediated in the inflammation pathway of doxorubicin-resistant HCC cells using human inflammatory antibody array membrane. Our array results indicated a reduction in most inflammatory mediators with an observed fold change between cells treated with 13.4 mM of melatonin, and control cells treated with DMSO. In several experimental inflammation models, melatonin was found to attenuate the production levels of inflammatory cytokines including TNF-α (tumor necrosis factor-α), IL-1β (interleukin-1β), IL-6, and IL-8. This action is probably mediated through a direct interaction with binding sites located in lymphocytes, and macrophages, or an indirect by blocking of the transcription factors that trigger pro-inflammatory cytokine production. All melatonin-inflammation studies concluded that melatonin may be a useful treatment for many inflammatory diseases and decreasing ROS formation within the cells (Bekyarova and Tzaneva 2015; Favero et al. 2017; Pahlavani et al. 2018; Hacışevki and Baba 2018; Wongsena et al. 2018; Schettig et al. 2020).

**Fig. 7:**
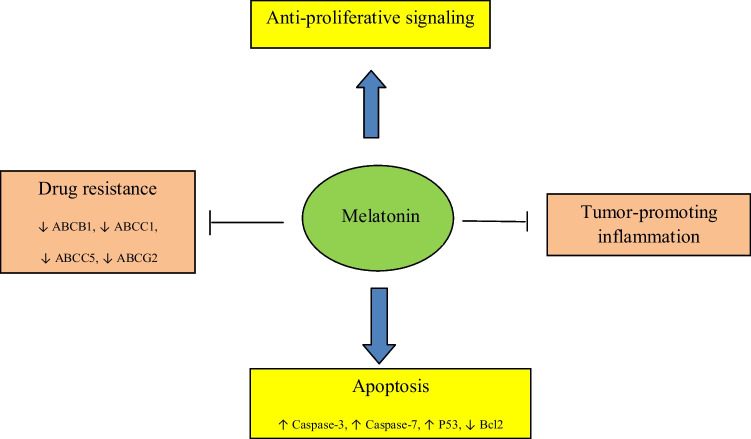
The summarized effect of melatonin on doxorubicin-resistant HepG2 cells used in the present study. ↑ = increase; ↓ = decrease

Through our data, we can summarize that melatonin enters into the doxorubicin-resistant HCC cells in a way that is independent of melatonin receptors and dependent on time, and dose manner. Then, it acts as an antioxidant to scavenge the generated ROS or activates the NRF2 cascade as a result of the elevation of doxorubicin-producing ROS. Subsequently, Melatonin could directly, or indirectly target the induced ABCs transporters that were expressed as an acquired resistance against doxorubicin elevated doses. Moreover, melatonin appears an activity against the inflammatory pathway-mediated molecules which provoke drug resistance too, and forces cells toward the apoptotic pathway, and cell cycle arrest (Fig. 7).

Referring to previous studies that were conducted in HCC-HepG2 cells, melatonin was reported to be administrated mainly as co-treatment with different studied doses of anti-cancer drugs, including tunicamycin (Zha et al. 2012), Cocl2 (Carbajo-Pescador et al. 2013), 1.25 mg/L doxorubicin (Fan et al. 2010), 10 µM cisplatin (Hao et al. 2017), sorafenib (Mortezaee 2018), combination of tunicamycin and 2.5 mg/L doxorubicin (Fan et al. 2013), and 20 µM cisplatin (Bennukul et al. 2014). All these studies confirmed the effective function of melatonin as an adjuvant therapy that can increase apoptosis (increase the cleavage of caspase-3, decrease the expression of Bcl2), decrease inflammation (reduce the expression of COX-2 [cyclooxygenase-2]), and decrease the cell viability. For exception, one study that has been applied 10^−5^ mM of melatonin for 24 h as post-treatment of HepG2 cells by 3 µM tunicamycin concluded that post-treatment by melatonin induces apoptosis via increasing the cleavage of caspase-3 (Bu et al. 2017).

## Conclusion

Collectively, we can conclude that for the first-time melatonin was applied as a treatment in a doxorubicin-resistant HepG2 cell model to relieve the bad side effects of chemotherapy. So, it may be a promising safe anti-cancer agent that will be administered individually, as adjuvant or post-chemotherapy treatment to improve the life quality of HCC patients with resistant tumor cells. As it is the first melatonin study conducted in resistant HCC cells, we recommend further wide studies.

## Supplementary Information

Below is the link to the electronic supplementary material.

Supplementary file1 (XLSX 11 KB)

Supplementary file2 (PZF 187 KB)

Supplementary file3 (PZF 169 KB)

Supplementary file4 (XLSX 22 KB)

Supplementary file5 (XLSX 21 KB)

Supplementary file6 (PZFX 12 KB)

## Data Availability

All are available in the current research paper.
